# Absolute Quantitation of Phenolic Compounds in Olive Oil for Health Claim Recognition

**DOI:** 10.3390/antiox15040511

**Published:** 2026-04-20

**Authors:** Ana Castillo-Luna, Feliciano Priego-Capote

**Affiliations:** 1 Department of Analytical Chemistry, Campus of Rabanales, University of Córdoba, 14014 Cordoba, Spain; 2 Chemical Institute for Energy and Environment (IQUEMA), Campus of Rabanales, University of Córdoba, 14014 Cordoba, Spain; 3 Maimónides Institute Biomedical Research (IMIBIC), Reina Sofía University Hospital, University of Córdoba, 14004 Cordoba, Spain; 4 Consortium for Biomedical Research in Frailty & Healthy Ageing, CIBERFES, Carlos III Institute of Health, 28029 Madrid, Spain

**Keywords:** olive oil, phenols, health claim, LC–MS/MS, gold standard, secoiridoids

## Abstract

The European Regulation (UE) 432/2012 includes a specific health claim for olive-oil-associated with its phenolic content, which is based on its protective role against lipid oxidation in the blood. To make use of the health claim, olive oil must have a minimum concentration in phenolic compounds of 250 mg/kg. Reviewing the health claim, the phenolic compounds referred to are the secoiridoid derivatives of hydroxytyrosol and tyrosol. A method based on absolute quantification of phenolic compounds in olive oil is proposed for the recognition of the health claim. The method involves liquid–liquid extraction with a 1:8 (*v*/*v*) oil:extract ratio to avoid saturation of the extract in oils with a higher phenolic content and its subsequent determination through LC–MS/MS in multiple reaction monitoring (MRM) mode, the gold standard technique in many application fields because of its analytical features. The optimized method was applied to a set of 100 extra virgin olive oils (EVOOs), and the results obtained were compared with the classic Folin–Ciocalteu method. The comparison between the two methods showed that the classic method is a non-selective method that can be affected by many interferences and that the Folin method underestimates the real phenolic content.

## 1. Introduction

Currently, the extra virgin olive oil (EVOO) market is being influenced by consumer demands that prioritize specific attributes providing added value to the product. Notable among these are certified quality, sensory profile, sustainability, and ethics, as well as health and nutritional value [1,2,3]. This latter aspect is directly associated with the health benefits of daily EVOO consumption, which are attributed to its monounsaturated profile and, fundamentally, its phenolic content, given the protective role of these compounds against oxidative stress [4]. It is precisely this phenolic fraction that is responsible for the only health claim in the European Regulation (UE) 432/2012, specific to olive oil. This claim states that the regular consumption of an olive oil, providing 5 mg of phenolic compounds via a recommended daily intake of 20 g, offers a beneficial effect by protecting blood lipids against oxidative damage. When translated into an absolute concentration, this is equivalent to a phenolic content of 250 mg/kg [5].

The health claim defines phenolic compounds as hydroxytyrosol and its derivatives, emphasizing oleuropein and tyrosol complexes. Numerous studies characterizing olive oil have identified secoiridoid derivatives of oleuropein and ligstroside as the predominant phenols. Prominent among these are the aglycone isomers of oleuropein and ligstroside, which can exist in monoaldehydic and dialdehydic forms, also described as closed and open isomers. These compounds are commonly referred to as oleuropein aglycone and ligstroside aglycone for the monoaldehydic closed forms, while oleomissional and oleokoronal are the names proposed for the dialdehydic open isomers of oleuropein and ligstroside aglycone, respectively [6,7]. These compounds are produced during fruit ripening and the oil extraction process through the action of the enzyme β-glucosidase. Furthermore, these compounds serve as precursors to the decarboxymethylated forms of oleuropein and ligstroside aglycone, commonly known as oleacein and oleocanthal. The formation of these compounds involves the enzyme esterase, which primarily utilizes the aforementioned open forms as substrates. Finally, the simple phenols, hydroxytyrosol and tyrosol, are derived from the hydrolysis of the described secoiridoid derivatives [8]. Although secoiridoid derivatives are not explicitly named in the health claim, they possess the highest antioxidant activity and are responsible for the health-promoting value of EVOO [9]. The fact that the health claim refers to hydroxytyrosol and its derivatives is explained by the technicality that most of these compounds were chemically characterized after the publication of the European Regulation 432/2012. In addition to these phenols, the presence of other phenolic families, such as lignans, flavonoids, and phenolic acids, has been described [10]; however, the health claim does not mention these groups.

Various methods have been proposed by official organizations for the quantitative analysis of phenolic compounds in olive oil. Remarkable among these are the Folin–Ciocalteu method and the methods proposed by the IOC (International Olive Council) based on liquid chromatography with photometric detection. The Folin–Ciocalteu method relies on a derivatization process involving oxidation with a reagent of a specific formulation and the photometric detection of oxidized complexes at 765 nm [11]. The primary limitation of this method is that any other compound with an oxidizable structure acts as an interference. Furthermore, the quantification is relative and highly dependent on the calibration model used. Common examples include solutions of caffeic, ascorbic, ferulic, or gallic acid, among others. The dependence on the specific molar response of the selected reference substance can lead to significant variations in the results, which can result in overestimation or underestimation of the actual phenolic content. Consequently, these factors explain why this method is not an appropriate method for addressing the health claim of the European Regulation. Regarding the IOC methods, the initial version is also not applicable to the health claim, as it expresses concentration results in terms of tyrosol content, which has been shown to cause an underestimation of the actual phenolic compound content. Recently, the second strategy has been proposed based on SPE and liquid chromatography (LC) with photometric detection to avoid the saturation effect that occurs during the liquid–liquid extraction stage. Additionally, this proposal incorporates an individual quantification strategy for each compound based on relative response factors relative to two standards: p-hydroxyphenylacetic acid and o-coumaric acid. This methodology would allow for the quantification of the content of each phenolic compound expressed in mg/kg or mmol/kg, although the methodological description itself specifies that the method would not be valid for concentrations exceeding 800 mg/kg. Despite the applicability of LC-based methods with photometric detection, selectivity is compromised due to the use of relative response factors and photometric monitoring at several wavelengths as the main criteria for the identification of compounds [12].

For this reason, the development of high selectivity methods preferentially based on the gold standard detection technique, LC coupled to tandem mass spectrometry (MS/MS), is desired. Despite LC–MS/MS having been widely used for determination of phenolic compounds in olive oil [13,14,15], an absolute quantitative method has not been optimized for the assessment of the health claim. With these premises, we propose a quantitative method based on LC–MS/MS for the determination of phenols in olive oil. The key aspects to be considered in this analytical development were: (i) the optimization of sample preparation through liquid–liquid extraction; (ii) the use of olive pomace oil for preparing calibration models; and (iii) the use of deuterated simple phenols as internal standards. The method was applied to a set of 100 EVOOs to compare results with those provided by the Folin–Ciocalteu method.

## 2. Materials and Methods

### 2.1. Chemicals and Reagents

LC–MS grade methanol (MeOH) and n-hexane, both from Fischer Scientific (Hampton, NH, USA), as well as deionized water (18 MΩ·cm) from a Milli-Q water purification system (Merck Millipore, Bedford, MA, USA), were used as solvents in the extraction process. LC–MS-grade acetonitrile (ACN) acquired from Fischer Scientific (Hampton, NH, USA) and deionized water were used to prepare the chromatographic phases. Ammonium acetate (1 M) from Scharlab (Barcelona, Spain) was used as an ionizing agent.

Analytical standards for individual phenols were purchased: simple phenols hydroxytyrosol and tyrosol from Extrasynthese (Genay, France), oleacein from TargetMol Chemicals (Boston, MA, USA), oleocanthal from Glentham Life Sciences (Corsham, Wiltshire, UK), oleuropein aglycone from Chengdu Biopurify Phytochemicals Ltd. (Chengdu, China), and ligstroside aglycone from TLC Pharmaceutical Standards (Newmarket, ON, Canada). Hydroxtyrosol-d_4_ and tyrosol-d_4_, which were used as internal standards (ISs), were acquired from Toronto Research Chemicals (Toronto, ON, Canada). Naringenin, used for quality control, was acquired from Sigma-Aldrich (St. Louis, MO, USA).

Olive pomace oil, characterized by a residual phenolic content and an EVOO selected by a low phenolic content were acquired from a local supermarket. Both products were used to determine the analytical features of the method.

Sodium carbonate (Na_2_CO_3_) was purchased from ITW Reagents Panreac (Barcelona, Spain), while caffeic acid and the Folin–Ciocalteu reagent were obtained from Merck (Darmstadt, Germany) and Scharlab (Barcelona, Spain), respectively. These chemicals were of analytical grade.

### 2.2. Instruments and Apparatuses

Liquid–liquid extraction of phenols from olive oil samples was carried out with a vortex shaker from IKA^®^ (Wilmington, NC, USA) and a Digitor 21 centrifuge from Orto Alresa (Madrid, Spain).

The analytes were separated through reversed-phase liquid chromatography using a Thermo Scientific UltiMate 3000 series LC system coupled with a Thermo Scientific QqQ TSQ QuantumTM Access MAX detector (Waltham, MA, USA) equipped with a ZORBAX RRHD Eclipse Plus C18 analytical column (1.8 µm particle size, 3.0 × 100 mm i.d.) preceded by a ZORBAX RRHD Eclipse Plus C18 guard column (1.8 µm particle size, 3.0 × 5 mm i.d.). Both columns were purchased from Agilent Technologies (Palo Alto, CA, USA), and the temperature was kept at 20 °C during the analysis.

Dionex Chromatography Mass Spectrometry Link (version 2.14.0.3818, ThermoScienfitic, Waltham, MA, USA) and Thermo TSQ Tune Master (version 2.5.0.1307, ThermoScienfitic, Waltham, MA, USA) were used to control the LC–MS/MS system. The method and worklist were generated using the Thermo Xcalibur software (version 3.0.63.3, ThermoScienfitic, Waltham, MA, USA), while data acquisition and qualitative and quantitative analyses were conducted using Thermo TraceFinder (version 3.2.368.22, ThermoScienfitic, Waltham, MA, USA).

Total phenolic compounds (TPCs) were determined using the Folin–Ciocalteu colorimetric method. The samples were incubated in an Eppendorf ThermoMixer C (Eppendorf AG, Hamburg, Germany). Absorbance was measured at 765 nm using a Helios Gamma UV-Vis spectrophotometer (Thermo Spectronic, Cambridge, UK).

### 2.3. Sample Preparation

A 0.5 g aliquot of olive oil was weighed. Then, 250 µL of hexane was added and was shaken for about 30 s to decrease the viscosity. Posteriorly, a dual liquid–liquid extraction with 2 mL of 80:20 MeOH:H_2_O containing the internal standards and quality control standard (1 μg/mL) was carried out for 3 min to isolate quantitatively phenolic compounds. The hydroalcoholic phases were separated by centrifugation at 900× *g* for 8 min and combined for further analysis by LC–MS/MS.

In the same vein, olive oil samples were analyzed using the Folin–Ciocalteu colorimetric method. First, 1 g of oil was weighed, 1 mL of hexane and 1 mL of MeOH:H_2_O were added, and the samples were shaken for 3 min to perform the initial liquid–liquid extraction. Posteriorly, the samples were centrifuged at 900× *g* for 8 min to separate the hydroalcoholic phase, after which a second liquid–liquid extraction was carried out with 0.5 mL of hexane, 0.5 mL of MeOH:H_2_O, and 2 min of shaking. After the centrifugation, both hydroalcoholic phases were combined and shaken for 1 min. Meanwhile, 1.6 mL of H_2_O and 0.3 mL of 20% *w*/*v* Na_2_CO_3_ were added to 20 µL of EVOO extract and 0.1 mL of Folin–Ciocalteu reagent. Then, the samples were incubated in the dark at 25 °C for 1 h in a ThermoMixer. Finally, the prepared samples were measured at 765 nm using a spectrophotometer.

### 2.4. LC–MS/MS Determination

Mobile phases contained 0.1% (*v*/*v*) ammonium acetate in deionized water (phase A) and in LC–MS grade acetonitrile (phase B). The chromatographic gradient was programmed with a constant flow rate of 0.3 mL/min, and the column was maintained at 20 °C throughout the analysis. The elution gradient was programmed as follows: it started at 30% B for 1.0 min, followed by a linear increase to 70% B from 1.0 to 3.5 min; this composition was held for 2.0 min, then ramped to 100% B at 6.0 min and maintained for 1.0 min to ensure the correct elution and column washing; finally, the system returned to the initial conditions (30% B) from 7.0 to 7.5 min, followed by a 4.5 min post-run for column re-equilibration. The total analysis time was 12 min. The injection volume was 5 µL.

The ion source parameters were optimized for maximum sensitivity: spray voltage, 2500 V; sheath gas pressure, 50 arbitrary units; auxiliary gas, 5 psi; vaporizer temperature, 100 °C; ion sweep gas pressure, 30 psi; and ion transfer capillary temperature, 250 °C. The ionization probe was set at position B on the y-axis and 0 on the x-axis. For MS detection, the peak width (FWHM) was set to 0.7 for both Q1 and Q3, while the scan time and width were 0.1 s and 0.5 *m*/*z*, respectively. The determination of phenolic compounds was carried out through multiple reaction monitoring (MRM, Table 1).

### 2.5. Quantitative Analysis of Phenols in EVOO Samples

Olive pomace oil was used for the calibration models. This oil was fortified with a multistandard phenolic solution, which contained oleacein, oleocanthal, hydroxytyrosol, tyrosol, oleuropein, and ligstroside aglycone (ranging from 0.8 to 40 mg/kg). All spiked aliquots were processed using the optimized protocol. Consequently, the extracts were measured through LC–MS/MS to obtain the calibration models for each individual phenol. The peak areas of oleuropein aglycone, oleacein, and hydroxytyrosol were normalized to the peak area of hydroxytyrosol-d_4_. By contrast, the peak areas of ligstroside aglycone, oleocanthal, and tyrosol were normalized to the peak area of tyrosol-d_4_ (Table 2). Naringenin was used as the quality control standard to monitor experimental variability. Additionally, the EVOO samples exceeding the upper limit of the calibration range were diluted 1:10 or 1:20 (*v*/*v*) and re-analyzed to ensure accurate quantification.

Furthermore, oleaceinic and oleocanthalic acid quantifications were carried out using the calibration models obtained from oleacein and oleocanthal, respectively. Similarly, the quantification of the monoaldehyde open isomers (oleokoronal and oleomissional) was performed using the calibration models obtained from the monoaldehyde closed isomers (ligstroside aglycone and oleuropein aglycone, respectively).

Relative quantification of the total phenolic content was performed using the Folin–Ciocalteu method with a calibration model (y = 0.001 ± 0.00003 + 0.035 ± 0.018), ranging from 50 to 900 mg/L (R^2^ = 0.9943). Following the protocols established in the reference literature for EVOO characterization, caffeic acid was used as the reference standard [15]. The results were expressed as caffeic acid equivalents (mg/L).

### 2.6. Applicability of the Proposed Method

The analytical applicability of the proposed method was evaluated by using a set of 100 EVOO samples produced in the 2024/2025 harvest season. These samples, which included monovarietal and blended oils from diverse geographical regions, were obtained from the EVOOLEUM World’s Top 100 EVOO Guide (2025 edition). These EVOOs were selected from a total number of 450 participants EVOOs after organoleptic analysis through the panel test method. The EVOOs were analyzed through the proposed method and the Folin–Ciocalteu method to compare results.

### 2.7. Data Treatment

Statistical analyses and data visualizations were performed using the R statistical software (version 4.4.3, http://www.r-project.org/). All analytical determinations were conducted in triplicate, and the results were expressed as mean values ± standard deviation (SD). Student’s *t*-test was performed using the stats package (version 0.1.0, https://cran.r-project.org/web/packages/pkgstats, accessed on 20 April 2026) to evaluate significant differences between the single and dual extraction protocols. The significance of the matrix effect was evaluated by comparing the slopes of the calibration models (prepared in deionized water and olive pomace oil) through analysis of covariance (ANCOVA) using the lm function from the stats package. To compare the efficiency of the developed LC–MS/MS method against the Folin–Ciocalteu (F-C) assay, the tidyverse (version 2.0.0, https://cran.r-project.org/web/packages/tidyverse, accessed on 20 April 2026) was used to facilitate a paired comparative analysis. The relationship between the two methods was examined further using Pearson correlation coefficients and linear regression models with the stat_cor function from the ggpubr package (version 0.6.2, https://cran.r-project.org/web/packages/ggpubr, accessed on 20 April 2026). Correlation plots and box plots were generated using the ggplot2 package (version 3.5.2, https://cran.r-project.org/web/packages/ggplot2, accessed on 20 April 2026).

## 3. Results and Discussion

### 3.1. LC–MS/MS for Determination of Phenols in Olive Oil

Phenolic determination in olive oil has been extensively used for characterization studies with different purposes. LC–MS/MS is considered the preferred technique for this determination because of its extraordinary analytical features that make this technique the gold standard for validation and confirmatory analysis [16,17]. The high sensitivity and selectivity of LC–MS/MS support its capability to determine phenols in olive oil.

Despite the analytical features of LC–MS/MS, the IOC methods for the determination of phenolic compounds are based on photometric detection by monitoring several wavelengths. According to the IOC declaration, only the second method can be applied to evaluate the health claim with a quantitative approach based on the use of response factors for the different phenolic compounds related to p-hydroxyphenylacetic acid and o-coumaric acid as internal standards [12].

Despite the fact that the phenolic family detected in olive oil includes numerous compounds, the studies carried out in recent years point out that major phenols found in olive oil are secoiridoid derivatives, which are structurally different to the mentioned internal standards [18,19]. These compounds are also responsible for crucial properties associated with virgin olive oil such as the bitterness and pungency attributes [20], the oxidative stability [21], and the health claim associated with their preventive role against oxidative stress. Secoiridoids in olive oil represent the main source of hydroxytyrosol and tyrosol derivatives, which are referred to in the EU 432/2012 regulation as hydroxytyrosol and derivatives, specifying oleuropein complex and tyrosol [5].

Phenolic determination is not a mandatory analysis according to regulatory and commercial standards, but its characterization is strongly considered to provide information about the quality of the product. For this reason, the method proposed in this research includes the most dominating secoiridoids, oleuropein aglycone, ligstroside aglycone, oleacein, and oleocanthal. In addition, the method allows the discrimination of the closed aglycone isomers, named oleuropein aglycone and ligstroside aglycone in this method, and the two open isomers, named oleomissional and oleokoronal in the literature. The method also includes simple phenols, tyrosol and hydroxytyrosol, which several studies have proposed as alteration markers due to the hydrolysis of secoiridoid structures. Two other markers, oleocanthalic acid and oleaceinic acid, are also monitored to detect chemical alterations associated with oxidation of oleocanthal and oleacein, respectively. Particularly, both acid derivatives have been proposed as ageing markers due to their increase in virgin olive oils with storage time [22,23]. Table 1 includes the main parameters for the optimal detection of these species, including an additional qualifier. The transitions from a precursor to product ions have been previously elucidated to avoid erroneous quantifications.

**Figure 1:**
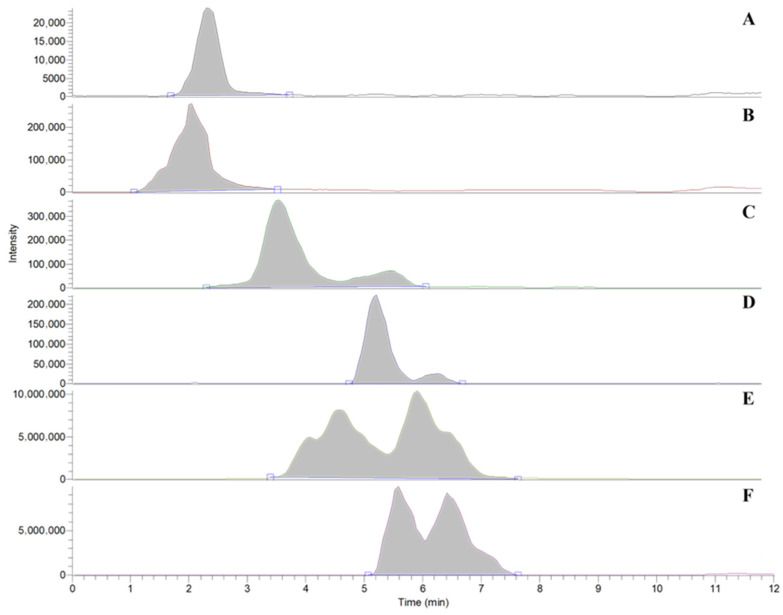
Representative LC–MS/MS chromatograms (Extracted Ion Chromatogram, EIC) showing the separation of targeted phenolic compounds in olive oil. Peak identification: (**A**): hydroxytyrosol; (**B**): tyrosol; (**C**): oleacein; (**D**): oleocanthal; (**E**): oleuropein aglycone; (**F**): ligstroside aglycone. Double peaks for compounds in (**E**,**F**) correspond to the isomeric forms.

Chromatographic separation of phenolic compounds has also been well defined. Gradients with acetonitrile or methanol are preferred. Although acetonitrile leads to a lower sensitivity compared to methanol, chromatographic resolution is better with the former, and sensitivity can be enhanced with the selection of a suited ionization agent. In this research, several agents were tested to evaluate the impact on the MS response, namely, acetic acid, formic acid, ammonium acetate, and ammonium fluoride. Special attention was paid to the test of ionization agents to tyrosol, which has been previously identified as the phenol with the lowest sensitivity due to the unique presence of an ionizable hydroxyl group. Ammonium acetate proved to be a suitable ionizing agent, and chromatographic separation was obtained in 8 min, as shown in Figure 1. Proof of the determination of oleaceinic acid and oleocanthalic acid is shown in Supplementary Figure S1. This proof was found in an EVOO that was analyzed 1 year after it was produced.

Sensitivity differences in the MS response of target phenols are evident when comparing calibration models obtained with the commercially available standards (Supplementary Figure S2). The slopes of the linear calibration models ranged from 90,674 ± 1425 to 4,384,140 ± 63,465, and the highest sensitivity of the MS detection response was obtained for oleuropein aglycone, while the lowest value was provided by tyrosol, which can be explained by the difference in the number of ionization capability of their functional groups.

### 3.2. Optimization of Sample Preparation

Sample preparation for phenolic analysis in olive oil has been mainly carried out through liquid–liquid extraction using methanol or acetonitrile solutions in water [24]. The efficiency of this extraction step is supported by the favoured solubility of phenols in the extractant compared to the non-polar oil. However, one limitation of this extraction approach would be a possible saturation of the extractant solution when phenolic concentration is particularly elevated. Quantitative studies have reported total phenolic concentrations above 1000 mg/kg [18]. For this reason, the IOC launched a second method for phenolic analysis based on SPE instead liquid–liquid extraction [12].

In this research, we planned an optimization study by evaluating three variables: the extractant composition, the number of extraction steps for quantitative isolation of phenols, and the extractant volume. In the first experiment, we evaluated the extractant composition and the number of extraction steps using 2 mL as volume. For this purpose, MeOH/water and ACN/water solutions, 60:40 and 80:20 (*v*/*v*), were tested using three consecutive extraction steps. The extraction efficiency for the first step with ACN/water was between 80% and 85% and between 79% and 86% for 60:40 and 80:20 (*v*/*v*) compositions, respectively. For MeOH/water as extractant, the efficiency ranged from 77% to 87% and from 80% to 88% with 60:40 and 80:20 (*v*/*v*) compositions, respectively. These results supported the application of the second extraction step due to a saturation effect in the first step. The combined use of the two steps allowed for reaching extraction efficiencies higher than 95% for the four extractant compositions, which ensured quantitative isolation of phenols from olive oil. No significant effects were found when analyzing the influence of the extractant composition through the application of the two consecutive extraction steps. However, chromatographic resolution was better if the isolation was carried out with MeOH/water solutions, particularly for separating oleuropein and ligstroside aglycone isomers. These results were obtained with a sample mass:total extractant volume of 1:8 (0.5 g/2 + 2 mL), which means that the extraction of phenols from olive oils in one single step must be avoided when the extractant volume:oil mass ratio is <8.

An additional study was conducted to determine whether the quantitative recovery of phenolic compounds achieved via dual extraction (2 × 2 mL) could be replicated using a single-step extraction with an equivalent total volume (4 mL). This comparison aimed to evaluate whether increasing the solvent-to-sample ratio in a single step is sufficient to displace the extraction equilibrium or if sequential steps are required for exhaustive recovery. Comparing the extraction results, efficiency was significantly (*p*-value < 0.01) higher for all phenols when a dual extraction step was applied (Table 3). Considering these results, a dual-step extraction process was adopted as the optimum for the sample preparation protocol.

### 3.3. Characterization of the Method

The method was characterized by the evaluation of analytical features such as the accuracy, sensitivity, and precision. Calibration models were prepared in pomace olive oil, which had previously been analyzed and reported to contain undetectable or trace levels of phenolic compounds. Olive pomace aliquots spiked with individual phenols in the concentration range of 0.8–40 mg/kg (0.1–5 μg/mL in the extracts) were subjected to analysis through the application of the optimized sample preparation protocol. Calibration models prepared in olive pomace oil were compared to those obtained in deionized water to evaluate matrix effects (Supplementary Table S1). Despite the fact that liquid–liquid extraction with hydroalcoholic phases allows obtaining clean extracts due to the non-polar composition of oil, matrix effects must be evaluated prior to determination of analytical features. The statistical comparison between slopes of calibration models prepared in pomace oil and water revealed significant differences (*p*-value < 0.001) in terms of sensitivity for all monitored phenols. For this reason, we proposed the use of olive pomace oil to prepare calibration models. In addition, we selected two internal standards to control the variability associated with the extraction step. The two standards were deuterated hydroxytyrosol and tyrosol, which were used in calibration models of secoiridoid derivatives according to their chemical structure. Thus, deuterated hydroxytyrosol was used for hydroxytyrosol-conjugated phenols (oleuropein aglycone and oleacein), whereas tyrosol was used for tyrosol-conjugated phenols (ligstroside aglycone and oleocanthal). The resulting calibration models are shown in Table 2. Regression coefficients were in all cases from 0.986 to 0.995, which proves a linear relationship between the analytical signal and concentration after the application of the complete process.

Sensitivity evaluation was complemented through the determination of detection and quantitation limits (LODs and LOQs, respectively) by following the IUPAC guidelines that define both limits according to the mean of blank measures and their standard deviations. LOQs were contrasted by considering the pure definition of this term, as the concentration derived from the smallest measure that can be detected with reasonable certainty through the application of the proposed analytical procedure [25]. Reasonable certainty was defined according to the variability ranges determined in the accuracy study. With these premises, LODs ranged from 5 µg/kg for oleuropein aglycone to 50 µg/kg for tyrosol, which reported the highest value. These values are considerably lower than phenolic concentrations typically found in VOOs, characterizing characterized this method as suitable for addressing the health claim that sets a cut-off value at 250 mg/kg.

Accuracy was evaluated through an analysis of an EVOO with low phenolic concentration (150 mg/kg) that was spiked with standard solutions at two concentration levels depending on the phenolic compound. Thus, hydroxytyrosol and tyrosol were spiked at 25 and 50 mg/kg, whereas secoiridoid derivatives were spiked at 50 and 100 mg/kg since they represent the most concentrated phenols found in VOOs. This experiment allowed for covering phenolic concentrations at 350 and 600 mg/kg at low and high spiking concentrations. Table 4 shows the accuracy results reported for each phenol, based on triplicate analysis of three aliquots prepared for each concentration level. These ranged from 71.0 to 90.6% and from 58.4 to 96.3% for simple phenols, hydroxytyrosol and tyrosol, respectively, and from 73.8 to 117% for secoiridoids. Nevertheless, simple phenols are minor phenols in olive oil. The results highlight the capability of the proposed quantitative method based on calibration models prepared in olive pomace oil with deuterated internal standards.

Finally, precision was determined through the implementation of a dual repeatability and reproducibility study dealing with the analysis of an EVOO with a high phenolic content around 1000 mg/kg. Within-day variability was evaluated through an analysis of three aliquots per day during 5 consecutive days. Variability was always below 13.5%. The lowest within-day variability was found for oleacein and oleocanthal, while the highest value was reported for ligstroside aglycone. Between-days variability was lower than 16.5%, with maximum values for oleomissional and ligstroside aglycone and the lowest values for hydroxytyrosol and oleocanthal (Table 5).

### 3.4. Application of the Method

The proposed method was applied to a set of 200 EVOOs produced in two consecutive agronomic seasons, 2023 and 2024. Extra virgin category was proved in all samples that were provided by the EVOOLEUM contest in the two editions. The analysis of these samples through the proposed method revealed a high variability in phenolic content in both agronomic seasons. Thus, EVOOs produced in 2023 reported a total phenolic content ranging from 79 to 1386 mg/kg, whereas in 2024, the concentration was ranging from 43 to 774 mg/kg. Mean phenolic concentrations were 421 and 343 mg/kg in the 2023 and 2024 editions, respectively. Considering the health claim concentration cut-off, 84 and 73% of 2023 and 2024 EVOOs, respectively, provided concentrations higher than 250 mg/kg and, therefore, could be recognized with the health claim established by the EFSA because of their preventive role against lipid oxidation in blood. Therefore, it is worth emphasizing that not all EVOOs, despite their extraordinary quality, contain sufficient phenolic concentration to qualify for the health claim. Oleocanthalic acid or oleaceinic acid were detected under quantitative limits in all monitored EVOOs.

Concerning the phenolic profiles, both editions were characterized by EVOOs with a common phenolic pattern, predominantly composed of hydroxytyrosol derivatives. Thus, 2023 EVOOs contained mean values of 255 and 166 mg/kg of hydroxytyrosol and tyrosol derivatives, respectively, and 2024 EVOOs contained 250 and 93 mg/kg, respectively. According to this contest, in both editions, premium EVOOs were characterized by a predominance of isomeric forms of oleuropein aglycone and oleacein.

**Figure 2:**
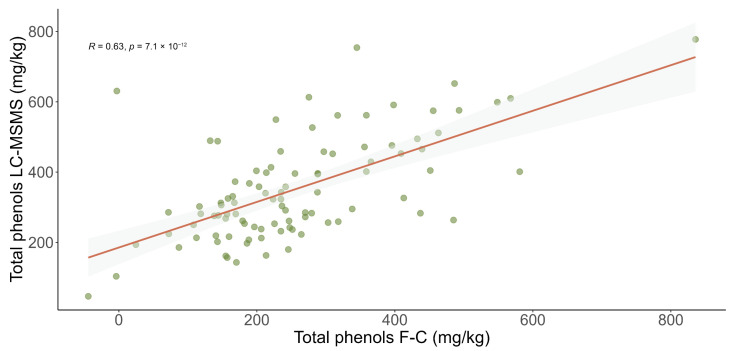
Correlation plot of total phenolic content (mg/kg) in EVOOs (Extra Virgin Olive Oil) from the 2024/2025 season: comparison between the optimized LC–MS/MS method (y-axis) and the Folin–Ciocalteu method (x-axis).

The 2024 edition EVOOs were also analyzed using the Folin–Ciocalteu method to compare the results with those provided by the proposed LC–MS/MS method. We found a significant correlation (R = 0.63, *p*-value < 0.001) between the total phenolic content provided by both methods (Figure 2).

**Figure 3:**
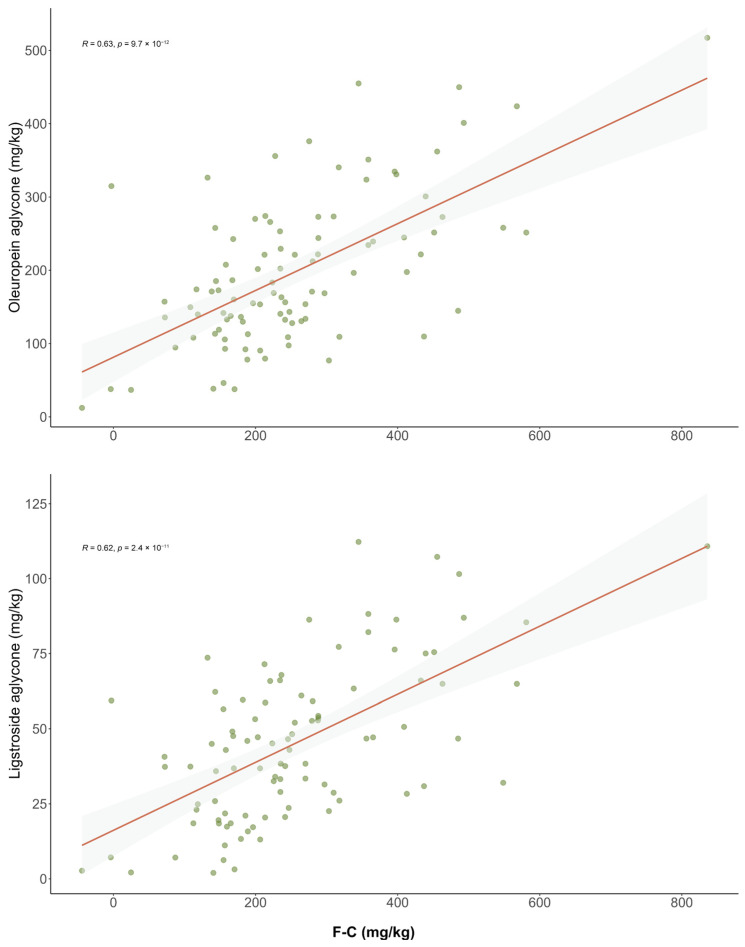
Correlation plots between the concentration of individual secoiridoids (mg/kg) determined by LC–MS/MS and the total phenolic content measured by the Folin–Ciocalteu (F-C) method in EVOOs.

Nevertheless, we found an underestimation effect in the results obtained by the Folin–Ciocalteu method since 81% of the samples reported higher phenolic content with the LC–MS/MS method. No specific patterns in the phenolic profiles allowed for explaining these differences. However, the Folin results provided significant correlations with the isomeric forms of oleuropein and ligstroside aglycones (R = 0.57 and R = 0.62, *p*-value < 0.001) (Figure 3), while no correlation was found with the sum of oleacein and oleocanthal, the two decarboxylated forms (R = 0.18) (Supplementary Figure S3). These correlations highlight the structural dependency of the colorimetric response of the primary phenols, which means that the Folin–Ciocalteu method may not accurately reflect the concentration of these phenols, justifying the use of more absolute methods such as LC–MS/MS.

**Figure 4:**
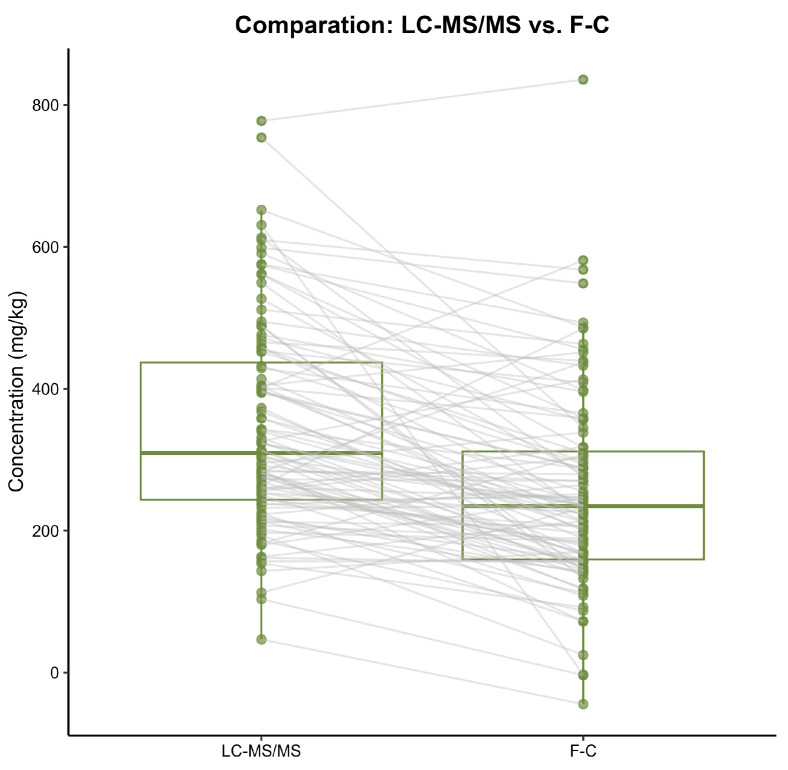
Box plot comparison of total phenolic content in EVOO samples by LC–MS/SM and Folin–Ciocalteu (F-C) methods.

Therefore, EVOOs with a moderate or high content in the last secoiridoid derivatives would provide an underestimation in the measurement by the Folin–Ciocalteu method. This effect was supported by the fact that 59% of the samples provided a total phenolic content measured using the Folin–Ciocalteu method lower than 250 mg/kg compared to the 26% obtained using the LC–MS/MS method (Figure 4). While underestimation using the Folin–Ciocalteu method does not pose a risk to consumer health, it creates a significant barrier for the olive oil industry. Due to the results obtained using the Folin–Ciocalteu method, EVOO producers who are close to the regulatory threshold may be unable to achieve the health claim established by the European Food Safety Authority (EFSA). Therefore, an absolute quantification by LC–MS/MS is crucial for an accurate determination of phenols as a differentiation criterion in the global EVOO market.

## 4. Conclusions

A method has been developed to quantitatively determine the presence of phenolic compounds in olive oil to recognize the health claim based on the protective role of these compounds against oxidative stress. This method involves a dual liquid–liquid extraction step, followed by further analysis of the extract using LC–MS/MS. The method has been characterized in terms of sensitivity, precision, and accuracy, providing quantitative measurements in absolute terms. Analysis of a set of extra virgin olive oils (EVOOs) using the proposed method and the Folin–Ciocalteu method revealed an underestimation bias in the response provided by the colorimetric method. In this regard, it is worth noting that the selection of alternative reference standards for the Folin–Ciocalteu method could yield results closer to LC–MS/MS due to the different molar responses. However, the main objective of this study was to establish a highly selective, sensitive, and accurate LC–MS/MS method. This ensures the absolute quantification of individual and total phenolic compounds, which is essential for the attribution of the health claim established by the EFSA.

## Figures and Tables

**Table 1:** LC–MS/MS parameters for determination of phenols.

Compound	RT * (min)	Precursor Ion (*m*/*z*)	Product Ion (*m*/*z*)	Tube Lens (V)	Collision Energy (eV)
Oleaceinic acid	1.5	335.2	199.9 **	90	30
			156.2		30
Oleocanthalic acid	1.5	319.5	181.1 **	85	25
			111.0		25
Hydroxytyrosol	1.9	153.2	123.3 **	79	16
			95.5		17
Hydroxytyrosol-d_4_	1.9	157.3	125.4 **	88	16
			96.5		25
Tyrosol	2.2	137.1	119.3 **	55	17
			106.3		19
Tyrosol-d_4_	2.2	141.3	108.6 **	95	18
			122.4		18
Oleacein	3.2	319.7	69.5 **	89	32
			249.8		11
Oleocanthal	5.0	303.3	69.3 **	85	30
			137.3		22
Oleuropein aglycone	5.6	377.5	275.8 **	91	14
			307.7		13
Ligstroside aglycone	6.4	361.6	291.7 **	78	17
			101.4		26
Naringenin	6.7	271.1	119.1 **	92	20
			151.1		20

* Retention time. ** Quantitation product ion.

**Table 2:** Calibration models prepared in olive pomace oil matrix for phenolic compound quantification.

Compound	Calibration Model	R^2^ *	Calibration Range
Hydroxytyrosol	y = 1.2761x ± 0.0342 − 0.1164 ± 0.0739	0.9865	0.8–40 mg/kg
Tyrosol	y = 0.4273x ± 0.0070 − 0.0345 ± 0.0151	0.9949	0.8–40 mg/kg
Oleacein	y = 0.1395x ± 0.0032 − 0.0070 ± 0.0068	0.9904	0.8–40 mg/kg
Oleocanthal	y = 0.8869x ± 0.0240 + 0.0032 ± 0.0518	0.9863	0.8–40 mg/kg
Oleuropein aglycone	y = 1.3651x ± 0.0344 − 0.0477 ± 0.0742	0.9881	0.8–40 mg/kg
Ligstroside aglycone	y = 2.0011x ± 0.0711 − 0.8518 ± 0.1657	0.9802	0.8–40 mg/kg

* Regression coefficient.

**Table 3:** Comparison of extraction efficiency (mg/kg) of phenolic compounds using single and dual extraction methods *.

Phenolic Compounds	Dual Extraction			Single Extraction	*p*-Value
	Step 1	Step 2	Total	Total	
Hydroxytyrosol	7.3 ± 0.16	1.9 ± 0.15	9.1 ± 0.31	8.1 ± 0.1	<0.001
Tyrosol	5.1 ± 0.51	0.76 ± 0.04	5.9 ± 0.55	4.3 ± 0.08	<0.01
Oleacein	131 ± 1.0	28.9 ± 4.1	160 ± 4.8	137 ± 1.2	<0.001
Oleocanthal	64.2 ± 0.61	16.8 ± 1.6	81.0 ± 0.95	75.3 ± 0.52	<0.01
Oleuropein aglycone	211 ± 0.89	48.4 ± 4.1	259 ± 3.2	248 ± 0.67	<0.001
Oleomissional	53.2 ± 0.34	10.8 ± 1.6	64.0 ± 2.0	48.6 ± 1.3	<0.001
Ligstroside aglycone	38.6 ± 0.77	10.6 ± 0.64	49.3 ± 0.14	52.4 ± 0.65	<0.001
Oleokoronal	12.1 ± 0.38	3.2 ± 0.6	15.3 ± 0.94	13.0 ± 0.27	<0.01

* *p*-values were calculated using Student’s *t*-test to compare total dual vs. single extraction.

**Table 4:** Accuracy assessment of the method proposed for quantification of phenolic compounds.

**Simple Phenols**	**25 mg/kg**			**50 mg/kg**		
	Test			Test		
	A	B	C	A	B	C
Hydroxytyrosol	79.1 ± 0.5	76.4 ± 0.2	71.0 ± 0.3	89.3 ± 1.4	89.7 ± 2.0	90.6 ± 1.4
Tyrosol	58.4 ± 6.0	60.0 ± 2.3	67.2 ± 4.4	89.7 ± 3.2	94.3 ± 3.8	96.3 ± 4.4
**Secoiridoids**	**50 mg/kg**			**100 mg/kg**		
	Test			Test		
	A	B	C	A	B	C
Oleacein	91.4 ± 1.0	92.3 ± 2.3	92.6 ± 2.1	115 ± 5.2	97.0 ± 1.1	90.7 ± 2.1
Oleocanthal	90.0 ± 1.8	88.9 ± 0.8	87.5 ± 1.3	117 ± 1.0	102 ± 0.3	105 ± 1.2
Oleuropein aglycone	73.7 ± 0.9	73.6 ± 0.8	73.8 ± 0.8	107 ± 1.9	99.2 ± 1.2	93.5 ± 0.6
Ligstroside aglycone	85.0 ± 0.7	84.9 ± 1.7	84.6 ± 2.6	113 ± 1.0	99.4 ± 2.5	96.4 ± 1.4

**Table 5:** Precision assessment of the method proposed for quantification of phenolic compounds.

	Within-Day Variability (RSD *, %, *n* = 5)	Between-Days Variability (RSD *, %)
Hydroxytyrosol	3.4 ± 1.3	5.6
Tyrosol	8.0 ± 2.9	15.6
Oleacein	2.4 ± 0.7	9.6
Oleocanthal	2.9 ± 0.6	5.4
Oleuropein aglycone	4.5 ± 2.4	10.7
Oleomissional	6.8 ± 4.6	16.3
Ligstroside aglycone	13.5 ± 1.2	16.5
Oleokoronal	4.6 ± 2.2	9.3

* Relative standard deviation.

## Data Availability

The data presented in this research are available in this article and in the Supplementary Materials.

## Supplementary Materials

The following supporting information can be downloaded at: https://www.mdpi.com/article/10.3390/antiox15040511/s1, Table S1: Calibration models prepared in deionized water for phenolic compound quantification. Figure S1: Representation LC–MS/MS chromatograms (EIC) showing the separation of targeted phenolic compounds in olive oil. Peak identification: A: oleaceinic acid; B: oleocanthalic acid. Figure S2: Representation of calibration models prepared in deionized water for phenolic compound quantification; Figure S3: Correlation plot between the combined concentration of oleacein and oleocanthal (mg/kg) measured by LC–MS/MS and total phenols by the Folin–Ciocalteu (F-C) method.

## References

[1] Sgroi F., Sciortino C., Giamporcaro G., Modica F. (2024). Exploring the Impact of Beliefs and Experiential Factors on Extra Virgin Olive Oil Consumption. J. Agric. Food Res..

[2] Kaliji S.A., Mater A., Universit S. (2025). Profiling Consumer Olive Oil Preferences: Shopping Behaviours, Health Orientations and Green Values. Br. Food J..

[3] Jiménez-Guerrero J.F., Gázquez-Abad J.C., Mondéjar-Jiménez J.A., Huertas-García R. (2012). Consumer Preferences for Olive-Oil Attributes: A Review of the Empirical Literature Using a Conjoint Approach. Olive Oil-Const. Qual. Health Prop. Bioconvers..

[4] Estruch R., Ros E., Salvadó J.S., Covas M., Corella D., Arós F., Gracia E.G., Gutiérrez V.R., Fiol M., Lapetra J. (2018). Primary Prevention of Cardiovascular Disease with a Mediterranean Diet Supplemented with Extra-Virgin Olive Oil or Nuts. New Engl. J. Med..

[5] (2012). Reglamento (UE) 432/2012 de Relativo a Las Declaraciones Nutricionales y de Propiedades Saludables en Los Alimentos.

[6] Miho H., Díez C.M., Mena-bravo A., De Medina V.S., Moral J., Melliou E., Magiatis P., Rallo L., Barranco D., Priego-Capote F. (2018). Cultivar influence on variability in olive oil phenolic profiles determined through an extensive germplasm survey. Food Chem..

[7] Servili M., Esposto S., Fabiani R., Urbani S., Taticchi A., Mariucci F., Selvaggini R., Montedoro G.F. (2009). Review Phenolic Compounds in Olive Oil: Antioxidant, Health and Organoleptic Activities According to Their Chemical Structure. Inflammopharmacology.

[8] Diamantakos P., Giannara T., Skarkou M., Melliou E., Magiatis P. (2020). Influence of Harvest Time and Malaxation Conditions on the Concentration of Individual Phenols in Extra Virgin Olive Oil Related to Its Healthy Properties. Molecules.

[9] Filardo S., Roberto M., Di D., Mosca L., Di M., Sessa R. (2024). Olea Europaea L—Derived Secoiridoids: Beneficial Health Effects and Potential Therapeutic Approaches. Pharmacol. Ther..

[10] de Torres A., Espínola F., Moya M., Alcalá S., Vidal A.M., Castro E. (2018). Assessment of Phenolic Compounds in Virgin Olive Oil by Response Surface Methodology with Particular Focus on Flavonoids and Lignans. LWT.

[11] Lamuela-ravents R.M. (1999). 2,6-Di-Tert-Butyl-4-Hydroxytoluene. **1999**, *299*, 152–178. Singleton, V.L.; Orthofer, R.; Lamuela-Raventós, R.M. Analysis of total phenols and other oxidation substrates and antioxidants by means of Folin-Ciocalteu reagent. Methods Enzymol..

[12] International Olive Council (IOC). https://www.internationaloliveoil.org/what-we-do/chemistry-standardisation-unit/standards-and-methods/.

[13] Dugo L., Russo M., Cacciola F., Mandolfino F., Salafia F., Vilmercati A., Fanali C., Casale M., De Gara L., Dugo P. (2020). Determination of the Phenol and Tocopherol Content in Italian High-Quality Extra-Virgin Olive Oils by Using LC-MS and Multivariate Data Analysis. Food Anal. Methods.

[14] Olmo-garcía L., Polari J.J., Li X., Bajoub A., Fernández-gutiérrez A., Wang S.C., Carrasco-pancorbo A. (2018). Deep Insight into the Minor Fraction of Virgin Olive Oil by Using LC-MS and GC-MS Multi-Class Methodologies. Food Chem..

[15] Olmo-garcía L., Fernández-fernández C., Hidalgo A., Vílchez P., Fernández-gutiérrez A., Marchal R., Carrasco-pancorbo A. (2019). Evaluating the Reliability of Specific and Global Methods to Assess the Phenolic Content of Virgin Olive Oil: Do They Drive to Equivalent Results?. J. Chromatogr. A.

[16] González-Domínguez R., Sayago A., Santos-Martín M., Fernández-Recamales A. (2022). High-Throughput Method for Wide-Coverage and Quantitative Phenolic Fingerprinting in Plant-Origin Foods and Urine Samples. J. Agric. Food Chem..

[17] Lucci P., Saurina J., Núñez O. (2017). Trends in LC-MS and LC-HRMS Analysis and Characterization of Polyphenols in Food. TrAC—Trends Anal. Chem..

[18] Criado-Navarro I., López-Bascón M.A., Priego-Capote F. (2020). Evaluating the Variability in the Phenolic Concentration of Extra Virgin Olive Oil According to the Commission Regulation (EU) 432/ 2012 Health Claim. J. Agric. Food Chem..

[19] Becerra-herrera M., Sánchez-astudillo M., Beltrán R., Sayago A. (2014). Determination of Phenolic Compounds in Olive Oil: New Method Based on Liquid e Liquid Micro Extraction and Ultra High Performance Liquid Chromatography-Triple e Quadrupole Mass Spectrometry. LWT—Food Sci. Technol..

[20] Tomé-Rodríguez S., Barba-Palomeque F., Calderón-Santiago M., Penco-Valenzuela J.M., Priego-Capote F. (2025). Phenolic Metabolism Explains Bitterness and Pungency of Extra. Foods.

[21] Miho H., Moral J., López-gonzález M.A., Díez C.M., Priego-Capote F. (2020). The Phenolic Profile of Virgin Olive Oil Is Influenced by Malaxation Conditions and Determines the Oxidative Stability. Food Chem..

[22] Tsolakou A., Diamantakos P., Kalaboki I., Mena-bravo A., Priego-capote F., Abdallah I.M., Kaddoumi A., Melliou E., Magiatis P. (2018). Oleocanthalic Acid, a Chemical Marker of Olive Oil Aging and Exposure to a High Storage Temperature with Potential Neuroprotective Activity. J. Agric. Food Chem..

[23] Angelis A., Antoniadi L., Stathopoulos P., Halabalaki M., Skaltsounis L.A. (2018). Oleocanthalic and Oleaceinic Acids: New Compounds from Extra Virgin Olive Oil (EVOO). Phytochem. Lett..

[24] Tasioula-margari M., Tsabolatidou E. (2015). Extraction, Separation, and Identification of Phenolic Compounds in Virgin Olive Oil by HPLC-DAD and HPLC-MS. Antioxidants.

[25] IUPAC “Limit of Detection” in IUPAC Compendium of Chemical Thermionology. https://goldbook.iupac.org/terms/view/L03540.

