# Supplementary material for: Absolute Quantitation of Phenolic Compounds in Olive Oil for Health Claim Recognition
Source: Antioxidants (Basel). 2026 Apr 20;15(4):511. doi: 10.3390/antiox15040511 (PMC13113915; doi:10.3390/antiox15040511)

Supplementary Table S1. Calibration models prepared in deionized water for phenolic compound quantification.

Compound	Calibration model	R^2*	Calibration range
Hydroxytyrosol	y = 3068536x ± 42795 – 75119 ± 92314	0.9963	0.1 – 5 mg/L
Tyrosol	y = 90674x ± 1425 – 256 ± 3075	0.9953	0.1 – 5 mg/L
Oleacein	y = 211020x ± 2192 – 13182 ± 4728	0.9904	0.1 – 5 mg/L
Oleocanthal	y = 141409x ± 2107 + 16654 ± 4545	0.9958	0.1 – 5 mg/L
Oleuropein aglycone	y = 4384140x ± 63465 – 554564 ± 136904	0.9960	0.1 – 5 mg/L
Ligstroside aglycone	y = 4589427x ± 121923 – 2106037 ± 284077	0.9888	0.1 – 5 mg/L

*Regression coefficient.

Supplementary Figure S1. Representation LC-MS/MS chromatograms (EIC) showing the separation of targeted phenolic compounds in olive oil. Peak identification: **A**: Oleaceinic acid; **B**: Oleocanthalic acid.

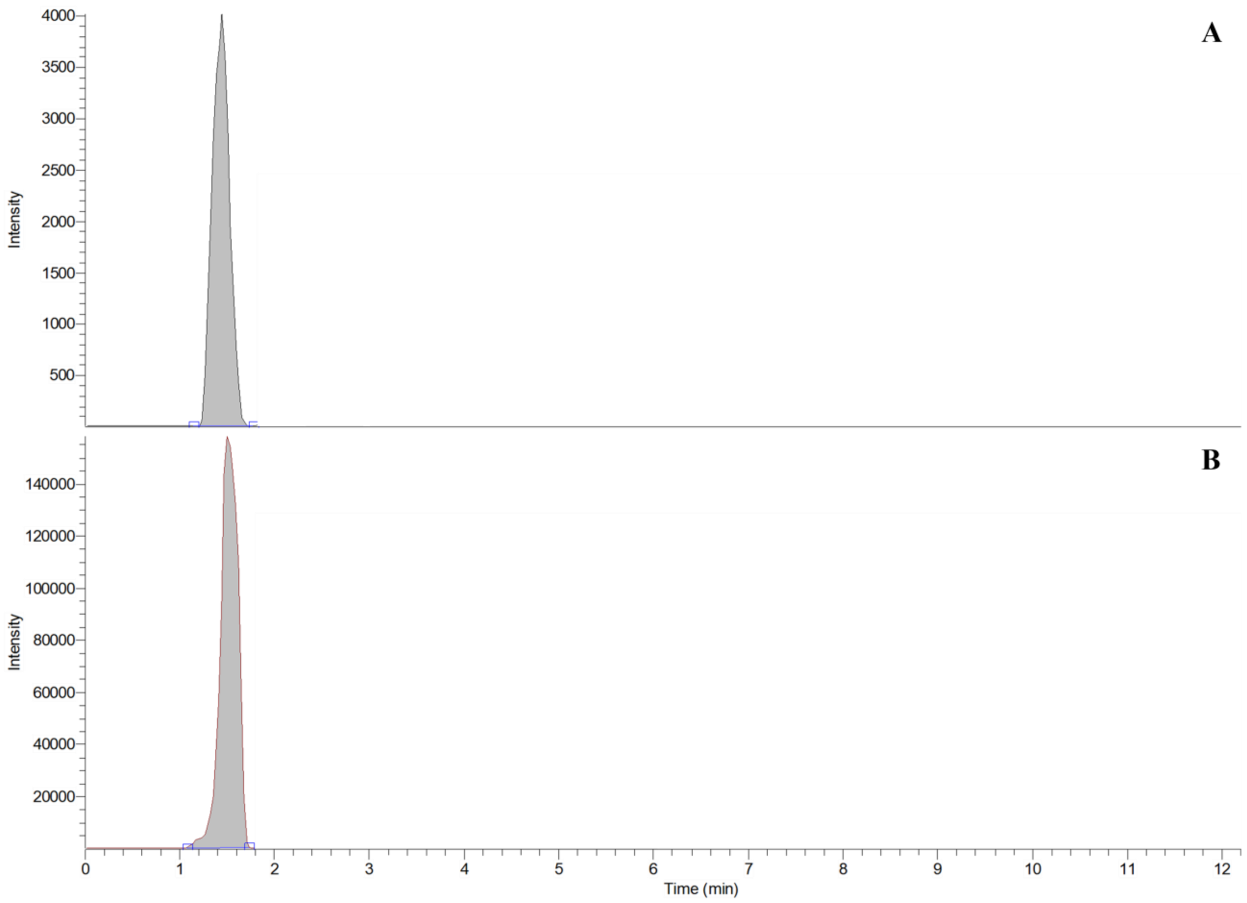

Supplementary Figure S2. Representation of calibration models prepared in deionized water for phenolic compound quantification.

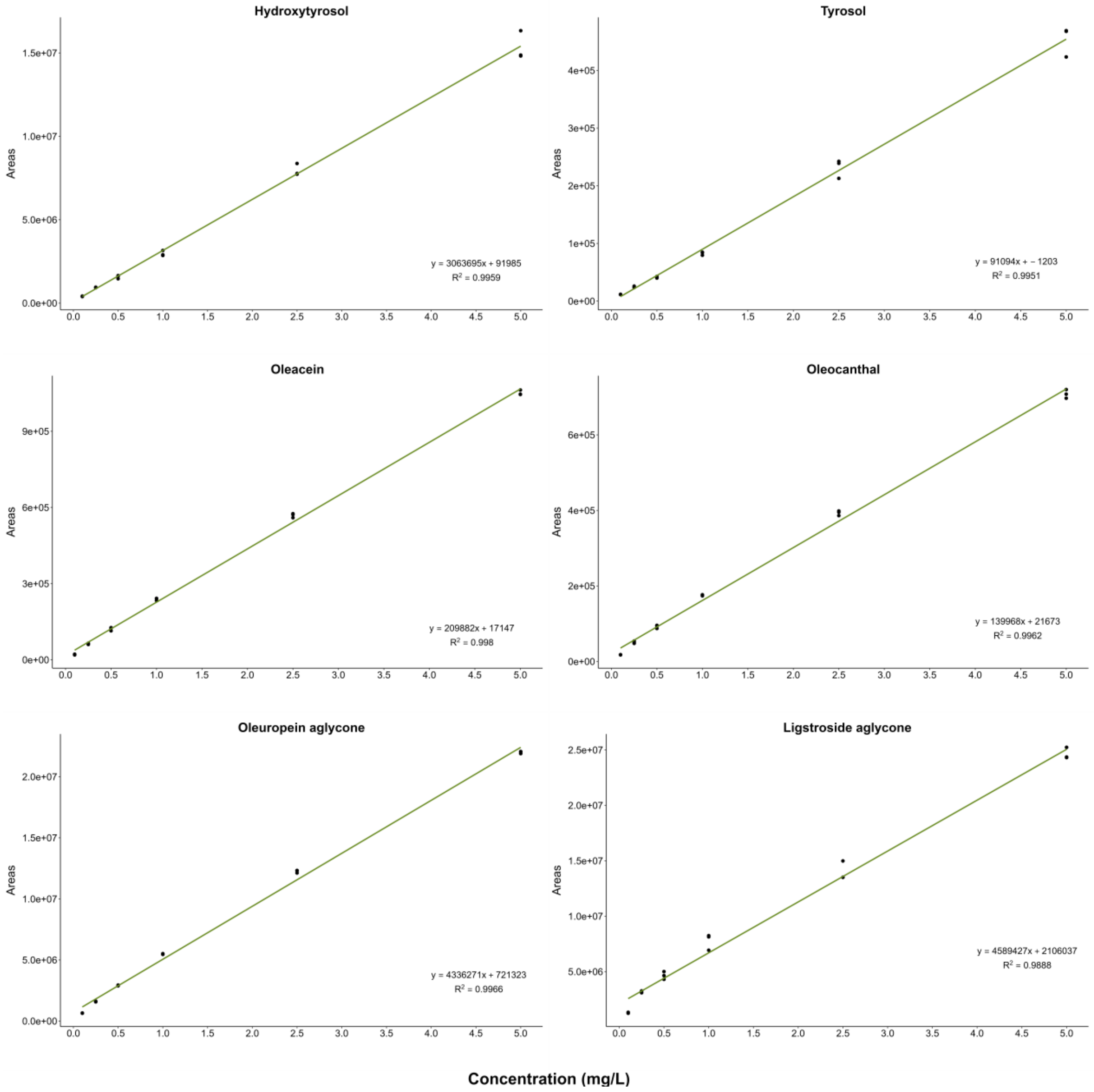

Supplementary Figure S3. Correlation plot between the combined concentration of oleacein and oleocanthal (mg/kg) measured by LC-MS/MS and total phenols by the Folin-Ciocalteu (F-C) method.

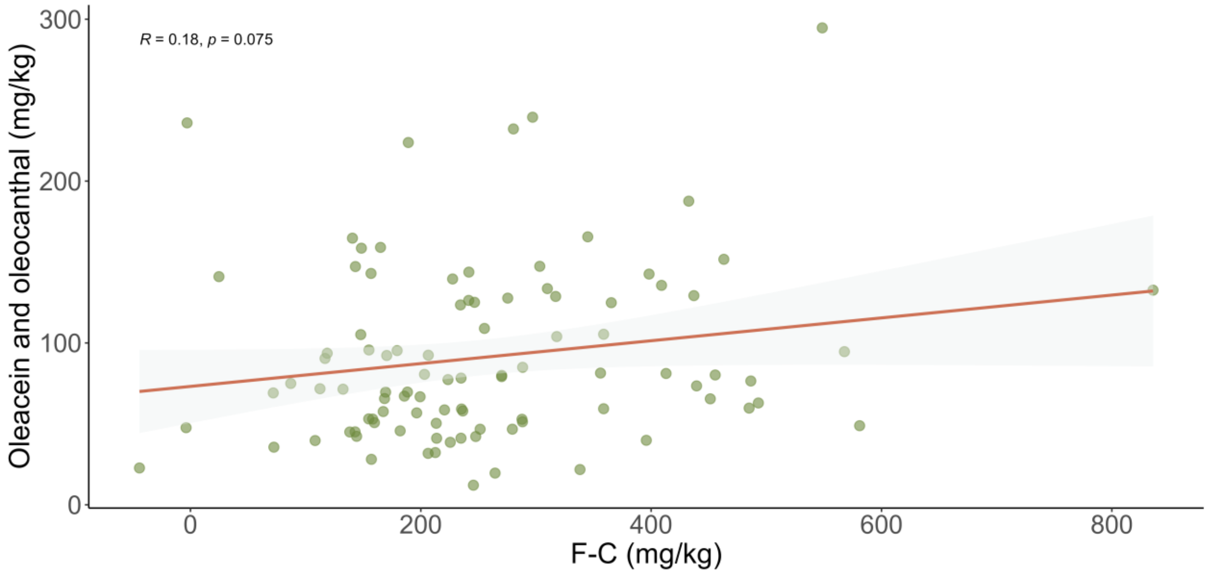

Supplement: Supplementary file 1 [file antioxidants-15-00511-s001.zip › antioxidants-4225978-supplementary.pdf]
